# The MADS-Box Gene *CsSHP* Participates in Fruit Maturation and Floral Organ Development in Cucumber

**DOI:** 10.3389/fpls.2019.01781

**Published:** 2020-02-10

**Authors:** Zhihua Cheng, Shibin Zhuo, Xiaofeng Liu, Gen Che, Zhongyi Wang, Ran Gu, Junjun Shen, Weiyuan Song, Zhaoyang Zhou, Deguo Han, Xiaolan Zhang

**Affiliations:** ^1^State Key Laboratories of Agrobiotechnology, Beijing Key Laboratory of Growth and Developmental Regulation for Protected Vegetable Crops, MOE Joint Laboratory for International Cooperation in Crop Molecular Breeding, China Agricultural University, Beijing, China; ^2^Key Laboratory of Biology and Genetic Improvement of Horticultural Crops of Northeast Region, Ministry of Agriculture, College of Horticulture & Landscape Architecture, Northeast Agricultural University, Harbin, China

**Keywords:** cucumber, *Cucumis sativus SHATTERPROOF*, floral organ identity, fruit maturation, abscisic acid

## Abstract

Cucumber is an important vegetable crop bearing fleshy pepo fruit harvested immature. Fruits left unpicked in time during summer production, as well as unfavorable environmental conditions during post-harvest shelf, will cause cucumber fruits to turn yellow and ripen, and thus impair the market value. Identification of maturity-related genes is of great agricultural and economic importance for cucumber production. Here, we isolated and characterized a MADS-box gene, *Cucumis sativus SHATTERPROOF* (*CsSHP*) in cucumber. Expression analysis indicated that *CsSHP* was specifically enriched in reproductive organs including stamens and carpels. Ectopic expression of *CsSHP* was unable to rescue the indehiscence silique phenotype of *shp1 shp2* mutant plant in *Arabidopsis*. Instead, overexpression of *CsSHP* resulted in early flowering, precocious phenotypes, and capelloid organs in wild-type *Arabidopsis*. Biochemical analysis indicated that CsSHP directly interacted with cucumber SEPALLATA (SEP) proteins. *CsSHP* expression increased significantly during the yellowing stage of cucumber ripening, and was induced by exogenous application of abscisic acid (ABA). Therefore, CsSHP may participate in fruit maturation through the ABA pathway and floral organ specification *via* interaction with CsSEPs to form protein complex in cucumber.

## Introduction

The fruit is a major evolutionary success in angiosperms which is essential for plant sexual reproduction and environmental adaptation (Fourquin and Ferrandiz, 2012). The main functions of fruits are to protect and nourish the developing seeds, and to act as a seed dispersal agent (van der Knaap et al., 2014). Angiosperms have evolved different types of fruit to meet the need of diverse dispersal strategies, such as the dry dehiscent fruit in *Arabidopsis thaliana* opens through dehiscence zones to release seeds, whereas the fleshy fruit in tomato attracts frugivorous animals to disperse seeds by means of bright color and pleasant aromas upon ripening (Roeder and Yanofsky, 2006; Ferrandiz and Fourquin, 2014). Moreover, fruits are the commercial organs for many agricultural crops and play important roles for human diet and health, thus the fruit has been under strong selective pressure during crop domestication (van der Knaap et al., 2014).

The fruit is generally developed from the ovary, which is an important component of the gynoecium. Gynoecium is located in the center of the flower, and surrounded sequentially as whorls by stamens, petals, and sepals, respectively (Robles and Pelaz, 2005). According to the ABC model, the sepal is specified by A function genes, the petal is determined by A+B function genes, the stamen is controlled by the B+C function genes, and the carpel is specified by the C class of genes (Coen and Meyerowitz, 1991; Weigel and Meyerowitz, 1994; Fourquin and Ferrandiz, 2012). In *Arabidopsis*, the *AGAMOUS* (*AG*) gene is the C class of gene that determines the carpel identity, specifies stamen identity with B-function genes, inhibits A-function genes and controls floral meristem determinacy (Bowman et al., 1989; Bowman et al., 1991; Mizukami and Ma, 1992; Mizukami and Ma, 1995). Subsequent studies showed that *SEPALLATA* (*SEP*) genes, expressing in four floral whorls, act as co-factors with ABC homeotic genes in specifying all types of ﬂoral organs (Theissen and Saedler, 2001; Favaro et al., 2003; Robles and Pelaz, 2005; Ruelens et al., 2017). Strikingly, all the genes involved in floral organ specification in *Arabidopsis* are from MADS-box transcription factor family excepting the gene *APETALA2* (Jofuku et al., 1994; Dreni and Kater, 2014). MADS-box genes are reported to be the key players in organ morphogenesis throughout the plant life cycle, with a typical MADS domain and a K-box domain in their protein structure (Smaczniak et al., 2012).

There are two MADS-box genes, the *SHATTERPROOF1 (SHP1)* and *SHP2*, in the AG subfamily of *Arabidopsis* acting as the major regulators directing dehiscence zone differentiation and stimulating lignification of adjacent cells in siliques (Liljegren et al., 2000). In the double mutant *shp1 shp2* plant, the mature siliques were unable to dehisce due to failure of dehiscence zone formation (Liljegren et al., 2000). Constitutive expression of *SHP* genes led to small fruits with overlignified valves (Liljegren et al., 2000). In other dry dehiscent fruits such as *Nicotiana benthamiana* (*NbSHP*), *Glycine max* (*GmAGL1*), and *Medicago*, the SHP gene promotes lignin accumulation in fruit pods to ensure cracking upon maturation (Liljegren et al., 2000; Fourquin and Ferrandiz, 2012; Fourquin et al., 2013; Chi et al., 2017). In the fleshy berry fruit tomato (Giovannoni, 2007), the *SHP1/2* ortholog *TAGL1* participates in fruit expansion and promotes fruit ripening. Knockdown of *TAGL1* resulted in yellow orange fruit with decreased carotenoids and thin pericarps. Overexpression of *TAGL1* led to enlarged sepals and overaccumulation of lycopene, supporting the roles of *TAGL1* in fruit ripening (Itkin et al., 2009; Vrebalov et al., 2009; Gimenez et al., 2010). Similarly, in the fleshy false fruit of strawberry, *FaSHP* was shown to promote maturation as well (Itkin et al., 2009; Vrebalov et al., 2009; Daminato et al., 2013). In citrus, CsMADS6 (the ortholog of SHP1/2) positively modulated carotenoid metabolism by directly regulating the expression of carotenogenic genes, suggesting an active role in fruit ripening (Lu et al., 2018). In addition to the functions in fruit opening and ripening, *SHP* genes play important roles in floral organ determination (Pinyopich et al., 2003; Vrebalov et al., 2009; Chi et al., 2017). In *Arabidopsis*, a redundant roles of *SHP1/2* and *AG* were found to promote carpel development, and overexpression of *SHP2* was sufﬁcient to rescue the stamen and carpel phenotypes in the *ag* mutant (stamens were replaced with petals and carpels were replaced by new abnormal flowers) (Bowman et al., 1989; Pinyopich et al., 2003). Transient knockdown of *NbSHP* in tobacco exhibited unfused pistils, and increased number of styles and stigmas (Fourquin and Ferrandiz, 2012). Ectopic expression of soybean *GmAGL1* in *Arabidopsis* resulted in petal-free flowers (Chi et al., 2017). Overexpression of grape *Vvmads1* (the ortholog of *SHP1/2*) in tobacco led to carpelloid sepals and stamenoid petals (Boss et al., 2001).

Cucumber (*Cucumis sativus* L.) is a world-wide cultivated vegetable crop bearing fleshy pepo fruit, which is developed from three-syncarpous inferior ovary (Che and Zhang, 2019). The fruit of North China cucumber can be generally divided into five developmental stages: immature green [0–9 days after anthesis days after anthesis (DAA)], breaker (12–15 DAA), turning (18–24 DAA), fully ripe (27–30 DAA), and senescence (35–43 DAA) (Leng et al., 2014). Cucumber fruits are consumed freshly or processed into pickles, and typically are harvested immature (about 7~14 DAA) (Weng et al., 2015). During summer production of cucumber, fruits left unpicked in time are prone to turn yellow and ripen on plants, which will greatly impair the commercial value and result in economic loss (Wang et al., 2013). Meanwhile, during cucumber post-harvest storage and transportation, unfavorable environmental conditions will cause immature fruits a series of senescence phenomena such as yellowing, accumulation of citrate, and tissue softening (Mainardi et al., 2006), which will adversely affect the market value. Therefore, identification of maturity-related genes is of great agricultural and economic importance for inhibiting fruit ripening on plants and delaying senescence of postharvest cucumbers.

As a non-climacteric fruit, it is abscisic acid (ABA), not ethylene, that was shown to promote fruit ripening on the cucumber plant (Nilsson, 2005; Hurr et al., 2009; Wang et al., 2013). To isolate putative fruit ripening related genes, we cloned a MADS-box gene *CsSHP* (the ortholog of *SHP*) in cucumber. *CsSHP* expression was highly enriched in reproductive organs of cucumber and were positively correlated with ABA accumulation during fruit maturation. Overexpression of *CsSHP* caused early flowering, precocious phenotypes, and ectopic capelloid organs in *Arabidopsis*. Biochemical analysis indicated that CsSHP directly interact with CsSEPs at protein level. Thus, CsSHP may participate in fruit maturation through the ABA pathway and floral organ specification *via* interaction with CsSEPs, which provides a possible target for genetic manipulation of fruit maturation progression to meet the different market demands in cucumber.

## Materials and Methods

### Plant Materials and Growth Conditions

Cucumber (*Cucumis sativus* L.) inbred lines R1461 (Chinese long type) was used in this study, and grown in the experimental field of China Agricultural University at Beijing under standard greenhouse conditions. The *A. thaliana Columbia* (*Col*) ecotype and the double mutant *shp1 shp2*, and relevant transgenic plants were grown in soil at 22°C under 16 h/8 h light/dark condition in a growth chamber.

### Sequence Alignment and Phylogenetic Analysis

A 711-bp fragment containing the complete *CsSHP* coding sequence was amplified from the young female buds using gene-speciﬁc primers (**Supplementary Table S2**). The gene structure of *CsSHP* was analyzed using online software GSDS 2.0 (http://gsds.cbi.pku.edu.cn/). Protein sequences of SHPs and other MADS-box genes from diverse plant species were obtained using the protein BLAST search (NCBI blast: https://blast.ncbi.nlm.nih.gov/Blast.cgi). Multiple sequence alignment was performed using CLUSTALW in MEGA5. The phylogenetic tree was generated using the neighbor-joining method in MEGA5 with 1,000 bootstrap replicates. The GenBank accession numbers for related proteins are listed in **Supplementary Table S1**.

### Quantitative Real-Time Polymerase Chain Reaction

The young leaves, stems, tendrils, male flower buds, female flower buds, male flowers, female flowers, ovaries at anthesis, fruits at different developmental stages from cucumber, as well as the inflorescences of *Arabidopsis* were frozen in the liquid nitrogen and stored at −80°C until use. Total RNA was extracted with TRIzol reagent as described in the manufacturer's instructions (Waryoung, China, http://www.huayueyang.com/), and cDNA was synthesized using FastQuant RT Kit (Tiangen, China, http://www.tiangen.com/). Quantitative real-time PCR (qRT-PCR) was performed using TB Green™ Premix Ex Taq™ (Takara, Japan, http://www.takarabiomed.com.cn/) on the Applied Biosystems 7500 RT-qPCR system. *Ubiquitin extension protein* (*UBI* CsaV3_5G031430) and *ACTIN2* (AT3G18780) were used as the internal reference genes in cucumber and *Arabidopsis*, respectively (Wan et al., 2010). The relative expression was calculated according to the comparative cycle threshold (C_T_) method (Schmittgen and Livak, 2008). Three biological replicates and three technical replicates were performed for each gene. The primer information is listed in **Supplementary Table S2**.

### *In Situ* Hybridization

The 25-day-old shoot tips, male and female ﬂower buds of R1461 were ﬁxed with 3.7% formalin-acetic acid-alcohol (FAA) fixative. Sampling and recognition of flower buds at different developmental stages were performed as described (Bai et al., 2004). Sense and antisense probes were ampliﬁed with gene-specific primers containing SP6 and T7 RNA polymerase binding sites, respectively. Sample ﬁxation, sectioning and hybridization were performed as described previously (Zhang et al., 2013). The primer information is listed in **Supplementary Table S2**.

### β-Glucuronidase Staining

β-glucuronidase (GUS) assay was performed according to the protocol as described previously (Liu et al., 2016). The *Arabidopsis* inflorescences and fruits at different developmental stage were ﬁxed and incubated in GUS-staining solution at 37°C for 24–48 h until dyed blue. Stained samples were then cleared with 70% ethanol and observed by anatomic microscope (Leica DFC450, Germany). The experiment was repeated three times.

### Subcellular Localization of *CsSHP*

The full-length CDS of *CsSHP* was fused into the pSUPER1300 vector to generate a CsSHP-GFP in-frame fusion protein. The empty pSUPER1300 vector was used as a positive control. *Agrobacterium tumefaciens* with the vectors were injected into the abaxial side of tobacco leaves (4–6 weeks old) by syringe as described previously (Schutze et al., 2009). After 48 h of inﬁltration, the ﬂuorescence of the expressed GFP proteins was detected and captured under 488 nm excitation wavelength from Argon laser of ﬂuorescence microscope (Leica sp5, Germany). The wavelength range for GFP is 495–545 nm.

### Ectopic Expression in *Arabidopsis*

The binary vector pBI121 and pSUPER1300 were used for ectopic expression of *CsSHP* in *Arabidopsis Col* and *shp1 shp2*, respectively. The *CsSHP* CDS was cloned into pBI121 and pSUPER1300 through *Xba*I and *Sma*I cleavages sites, and *Hind*III and *Kpn*I cleavages sites to generate *CsSHP* overexpression constructs, respectively. *Col* and *shp1 shp2* plants were transformed by *A. tumefaciens* containing the pBI121 and pSUPER1300 recombinant construct through the ﬂoral dip method, respectively (Clough and Bent, 1998). Transgenic seeds were germinated on solid Murashige and Skoog (MS) medium with 50mg/L kanamycin and 25mg/L hygromycin, respectively. Resistant seedlings were transferred to soil and further verified by PCR and qRT-PCR. Three biological replicates and three technical replicates were performed for each gene. T2 transgenic plants were chosen for further phenotypic analysis and data statistics. The primers used for vector construction and transgene identification are given in **Supplementary Table S2**.

### Measurement of Endogenous Hormones

About 0.3 g cucumber ovaries at different developmental stages were used as samples. The content of indole-3-acetic acid (IAA), zeatin riboside (ZR), abscisic acid (ABA) were measured using enzyme-linked immunosorbent assays according to methods previously described (Sun et al., 2016). Three biological replicates were performed.

### Abscisic Acid and Auxin Treatment

Three groups, each consisting of 20 cucumber ovaries at 4 days before anthesis, were used as samples. The first group was sprayed with a solution of 200 mg/L ABA in 0.5% (v/v) Tween 20, the second group was sprayed with a solution of 50 mg/L IAA in 0.5% (v/v) Tween 20, and the third group was sprayed only with 0.5% (v/v) Tween 20 as control (Daminato et al., 2013). Three ovaries from each group were collected after 0, 1, 3, 12, and 24 h treatments. RNA of the collected ovaries was extracted, and the *CsSHP* expression was evaluated by qRT-PCR.

### Yeast Two-Hybrid Assay

Full-length CDSs of *IND*, *SPT*, *CsSHP*, *CUM10*, *CsSEP2*, *CsSEP3*, and *CsSEP4* were cloned into pGADT7 (prey vector) or pGBKT7 (bait vector). All constructs were verified by sequencing and then transformed into yeast strain AH109. The yeast two-hybrid assays were conducted and protein interactions were analyzed on selective medium lacking Leu, Trp, His, and Ade (Ding et al., 2015). The primers for yeast two-hybrid assays are listed in **Supplementary Table S2**.

### Firefly Luciferase Complementation Imaging Assay

*CsSEP2*, *CsSEP3*, *CsSEP4*, and *CUM10* full-length CDSs without stop codon were cloned in pCAMBIA1300-nLUC, and *CsSHP* full-length CDS with stop codon in pCAMBIA1300-cLUC. *A. tumefaciens* GV3101 strain carrying the above constructs was mixed in proportion and resuspended, then injected into tobacco (*N. benthamiana*) leaves by syringe. The interactions of the expressed fusion proteins were indicated by reconstituted LUC enzyme after 2–3 days of inﬁltration, and images were obtained using a chemiluminescent imaging system (Tanon 5200, China) as described (Chen et al., 2008). The primers for vector construction are given in **Supplementary Table S2**.

## Results

### Identiﬁcation of *CsSHP* in Cucumber

**Figure 1:**
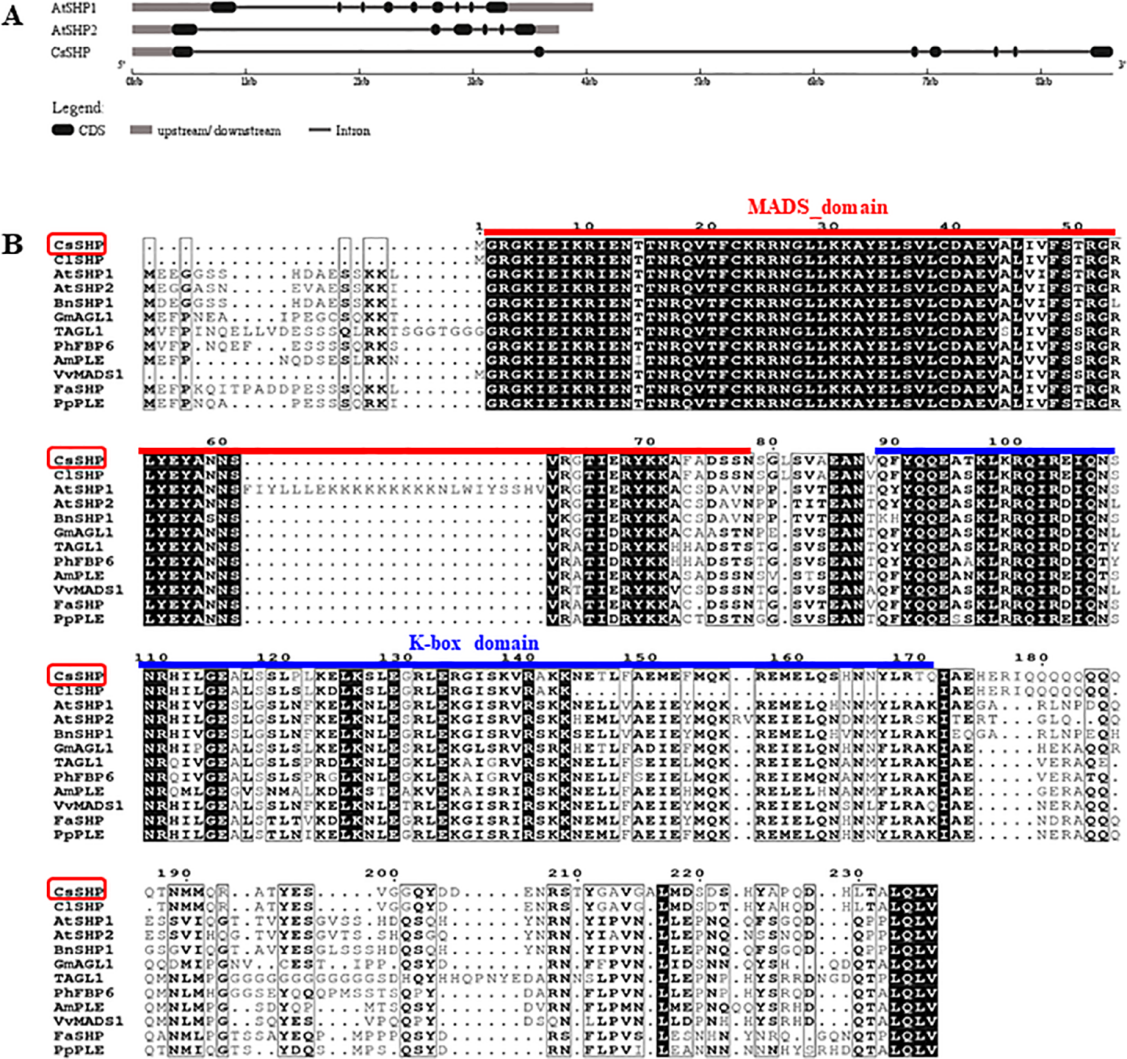
Gene structure and sequence alignment. **(A)** Gene structure analysis of *SHP1*, *SHP2*, and *CsSHP*. **(B)** Multiple sequence alignment of CsSHP and orthologs from related plant species. The red and blue lines indicate the conserved MADS domain and K-box domain, respectively.

To isolate the *SHP* gene in cucumber, we performed a BLAST search in National Center for Biotechnology Information (NCBI) database, and found the cucumber protein (NP_001292697.1) displays the highest sequence homology (59.7% identity) to *Arabidopsis* SHP1. A reciprocal BLAST search was performed in The *Arabidopsis* Information Resource (TAIR) and cucurbit genomics database (CuGenDB), and the NP_001292697.1 (CsaV3_6G015770.1) was confirmed to be the SHP homolog in cucumber, therefore, we named it as *CsSHP* hereinafter. There were two *SHP* genes (*SHP1* and *SHP2*) in *Arabidopsis*, while only one *SHP* in cucumber. The genomic sequence of *CsSHP* is 8,591 bp, which is much longer than that of *SHP1* (4,058 bp) and *SHP2* (3,759 bp) in *Arabidopsis*. *CsSHP* is predicted to contain seven exons and six introns, with the first and second intron being particularly long (3,005 and 3,232 bp, respectively) (**Figure 1A**). The second intron of AG/PLE genes contains several conserved motifs and has been shown to be essential for fulfilling their proper functions (Causier et al., 2009). The extremely large first and second introns in *CsSHP* may imply the more complex functional regulation than its *Arabidopsis* counterpart.

**Figure 2:**
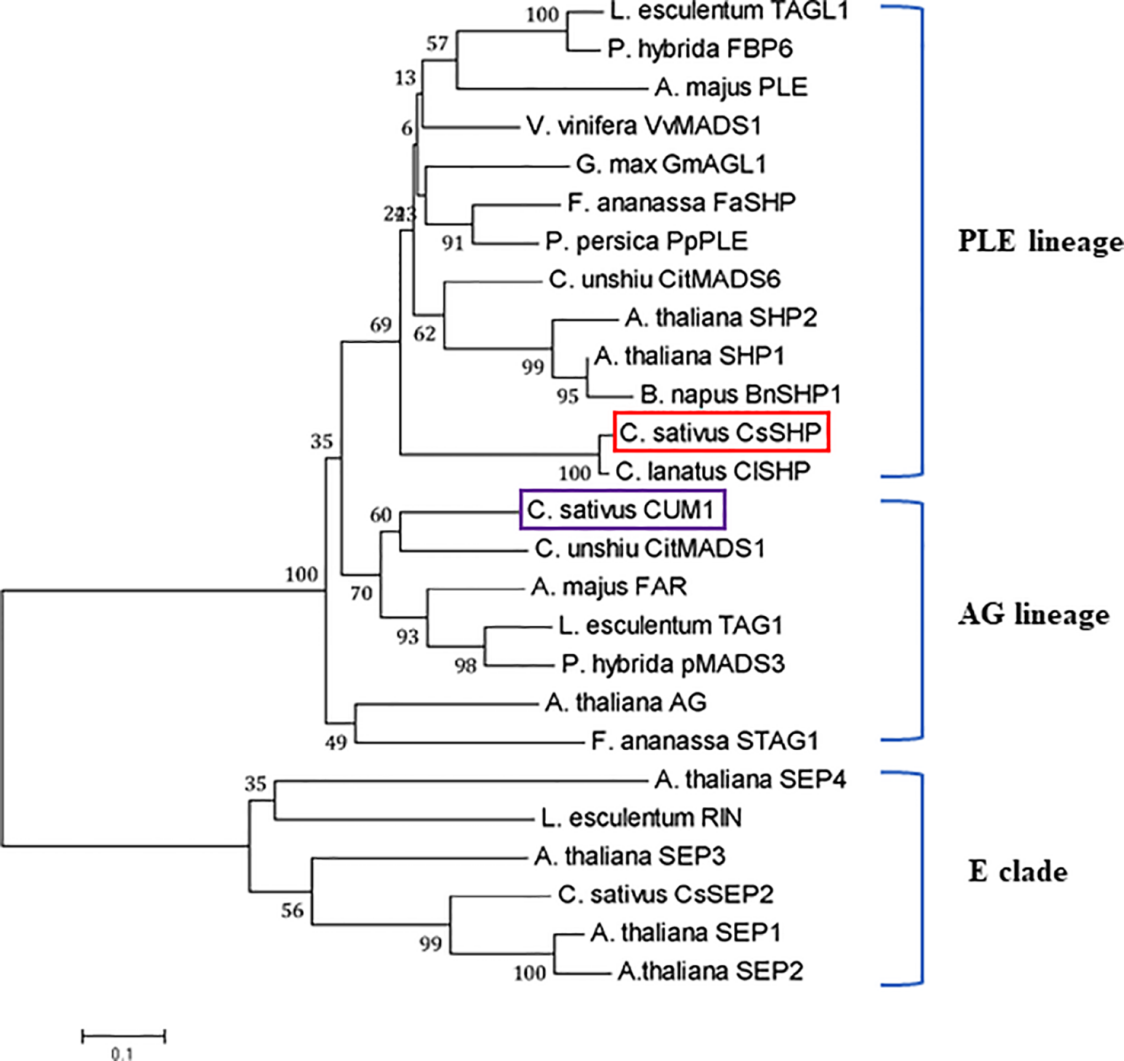
Phylogenetic analysis of CsSHP and its homologous proteins. The scale bar represents 0.1 substitutions per site. The red and purple squares indicate CsSHP and CUM1, respectively. E lineage proteins were used as outgroups.

The full-length coding sequence (CDS) of *CsSHP* was obtained from the female bud of cucumber inbred line R1461, which encodes a protein of 237 amino acids with a calculated molecular mass of 27.18 kD (**Supplementary S1**). A multiple sequence alignment of CsSHP and its homologs from other species indicated that these proteins contained the conserved MADS domain and K-box domain (**Figure 1B**). Phylogenetic analysis indicated that CsSHP is very close to ClSHP in watermelon, which was clustered with other known SHP proteins and located in the PLENA (PLE) lineages of AG subfamily, while CUM1 (the ortholog of AG in cucumber) was clustered with other AG proteins (**Figure 2**) (Kater et al., 1998).

### Expression Analysis and Protein Localization of *CsSHP*

**Figure 3:**
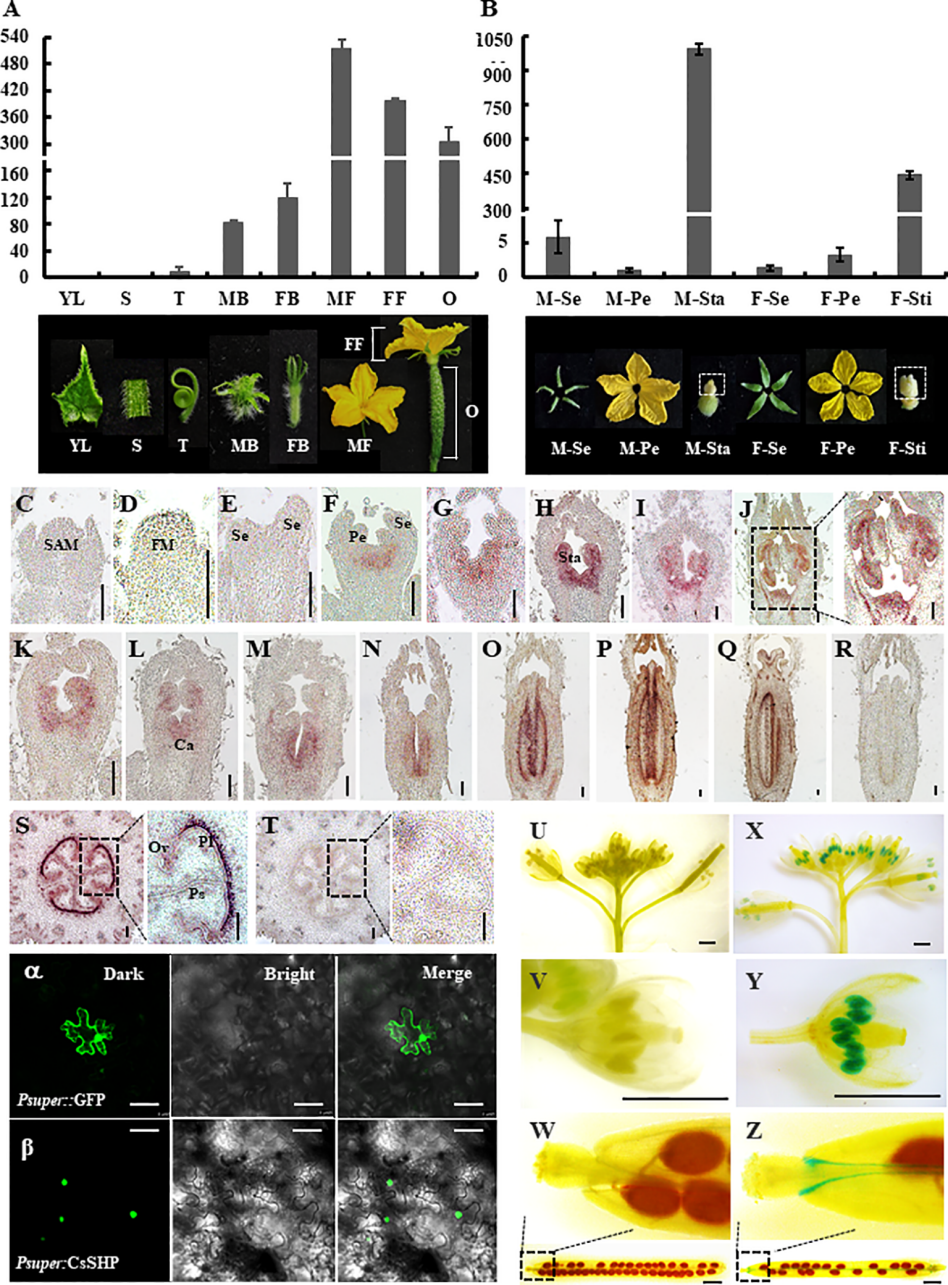
Expression analysis and subcellular localization of *CsSHP*. **(A)** quantitative real-time (qRT)-PCR analysis of *CsSHP* expression in different cucumber organs. YL, young leaves; S, stems; T, tendrils; MB, male buds; FB, female buds; MF, male ﬂowers at anthesis; FF, female ﬂowers at anthesis; O, ovaries at anthesis. Three biological replicates and three technical replicates were performed. **(B)** qRT-PCR analysis of *CsSHP* expression in different floral organs at anthesis. M-Se, sepals from male ﬂowers; M-Pe, petals from male ﬂowers; M-Sta, stamens from male ﬂowers; F-Se, sepals from female ﬂowers; F-Pe, petals from female ﬂowers; F-Sti, stigmas from female ﬂowers. Three biological replicates and three technical replicates were performed. Pictures of the corresponding organs are displayed at the bottom. **(C–T)** *In situ* hybridization of *CsSHP* in young organs of cucumber. Scale bar = 100 μm. **(C)** Shoot apical meristem (SAM). **(D–H)** Longitudinal sections of male flower buds at stages 1–5 in inbred line R1461. **(I, J)** Male flower buds at stages 8 and 9 in R1461 line. **(K–Q)** Longitudinal sections of female ﬂower buds at different developmental stages in inbred line R1461. **(S)** Transection of ovary 10 days before anthesis. **(R, T)** The sense *CsSHP* probe was hybridized as a negative control. FM, floral meristem; Se, sepal; Pe, petal; Sta, stamen; Ca, carpel; Pl, placenta; Ps, pseudoseptum; Ov, ovule. **(U–Z)** β-Glucuronidase (GUS) signals of *CsSHP* in *Arabidopsis*. Scale bar = 1 mm. **(U–W)** Negative control plants showed no GUS signal. **(X, Y)** GUS signals were highly enriched in stamens. **(Z)** GUS signals were detected in valve margin of silique. The experiment was repeated three times. **(α, β)** Subcellular localization of CsSHP. GFP signals were localized in the nucleus. GFP driven by the pSUPER promoter was used as a positive control. GFP is shown in green. The left, middle, and right panels represent pictures taken under dark ﬁeld, bright ﬁeld, and merge views, respectively. Scale bar = 50 μm.

To explore the expression pattern of *CsSHP*, qRT-PCR was performed in different cucumber organs including young leaves, stems, tendrils, male buds, female buds, male ﬂowers at anthesis, female ﬂowers, and ovaries at anthesis. Transcripts of *CsSHP* were highly accumulated in reproductive organs such as male ﬂowers, female ﬂowers, and ovaries, but with low levels in vegetative organs including leaves, stems, and tendrils (**Figure 3A**). In the four floral organs at anthesis, *CsSHP* was specifically expressed in stamens of male flowers and stigma of female flowers, while very low levels in sepals and petals (**Figure 3B**).

A previous study showed that *SHP1/2* in *Arabidopsis* was speciﬁcally expressed in gynoecia, but not in stamens (Pinyopich et al., 2003; Colombo et al., 2010). To further detect the spatial and temporal expression pattern of *CsSHP*, *in situ* hybridization was applied to the shoot apex, ovaries, and flower buds at different developmental stages in cucumber. In the shoot apical meristem (SAM), floral meristem, and sepal primordia, *CsSHP* transcripts were undetectable (**Figures 3C–E**). *CsSHP* transcripts were first found in the initiating stamen primordia at stage 3 (**Figure 3F**), then *CsSHP* was expressed at stamen and carpel primordia from stage 4 to 6 (**Figures 3G, H**), and maintained its expression in developing stamens and degenerate carpels in male flowers (**Figures 3I, J**). In female flower buds, *CsSHP* was highly expressed in carpel primordia and decreasingly expressed in degenerate stamens (**Figures 3K–N**). Since stage 8, *CsSHP* transcripts were specifically detected at stigmas, placenta, and ovule primordia (**Figures 3O–Q**). No signal was detected upon hybridized with the sense probe control (**Figure 3R**). Transverse sections of ovaries showed significant enrichment of *CsSHP* signals at placenta, pseudoseptum, and ovules (**Figures 3S, T**).

To visualize the expression pattern at whole plant level, transgenic *Arabidopsis* lines expressing β-glucuronidase (GUS) driven by the 1,716 bp *CsSHP* promoter fragment were generated. Unlike the specific expression of *SHP2* throughout the developing gynoecium in *Arabidopsis* (Colombo et al., 2010), GUS signal of *CsSHP* was found strongly in stamens as well as the valve margin of siliques, but not in developing gynoecium (**Figures 3U–Z**).

To explore the subcellular localization, CsSHP was fused with GFP under the control of a pSUPER promoter, and transiently expressed in tobacco leaves. Confocal green ﬂuorescence imaging revealed that CsSHP-GFP fusion protein was located to the nucleus, whereas free GFP was distributed throughout the cell (**Figures 3A, B**).

### Ectopic Expression of *CsSHP* Led to Early Flowering and Disturbed Floral Organ Development in *Arabidopsis*

**Figure 4:**
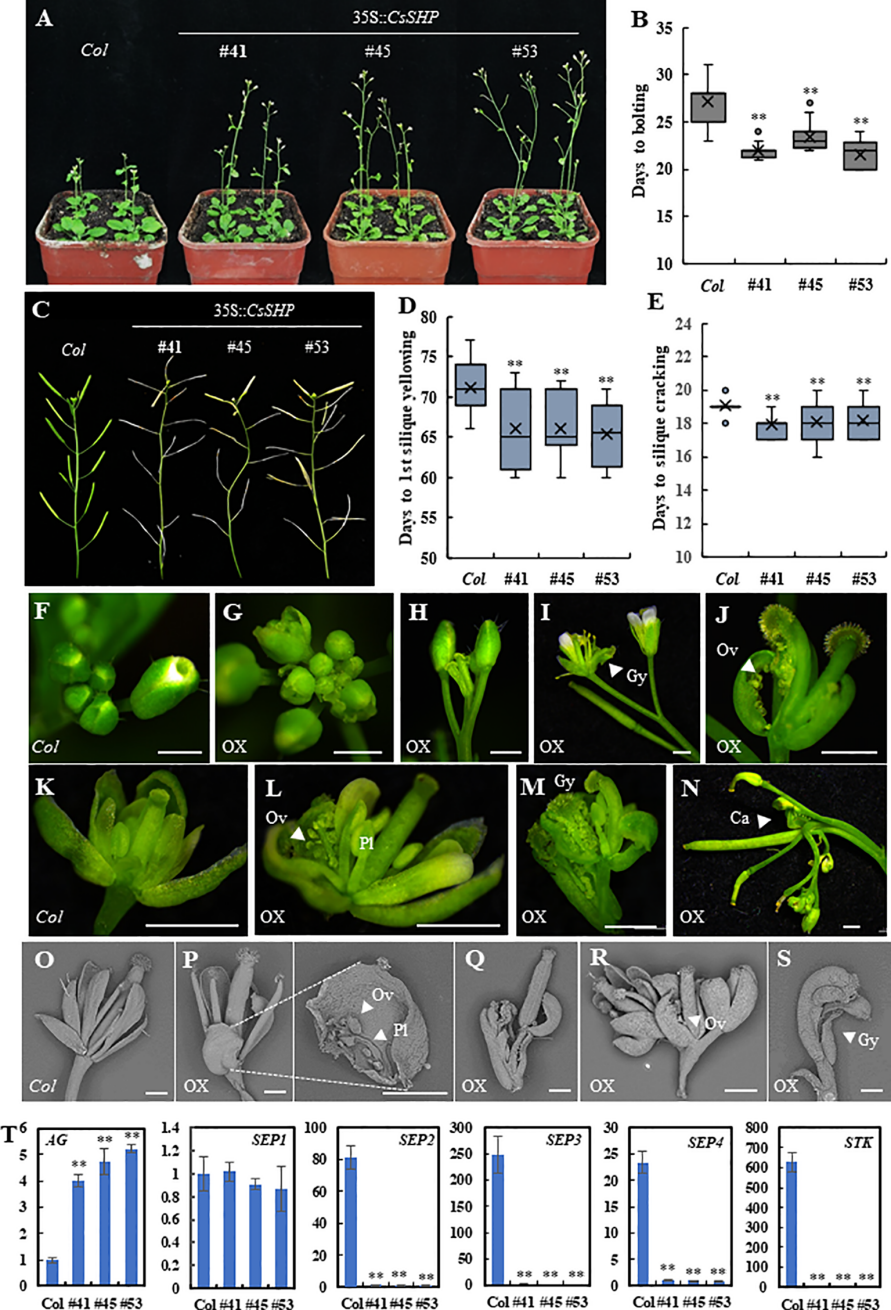
Phenotypic characterization of *35S::CsSHP* transgenic plants in *Arabidopsis*. **(A)** Representative images of *35S::CsSHP* transgenic plants indicated early flowering in *Arabidopsis*. **(B)** Box-plot of the days to bolting in *Col* and *35S::CsSHP* transgenic lines. **(C)** Overexpression of *CsSHP* led to precocious phenotype in *Arabidopsis*. **(D, E)** Quantification of the days to yellowing of the 1^st^ silique **(D)** and the days from anthesis to silique cracking **(E)**. The data were the average of 20 plants for each line. **(F–S)** Overexpression of *CsSHP* caused defected floral organ development in *Arabidopsis*. **(F–N)** Anatomic micrographs of flowers and inflorescences. Scale bar = 1 mm. **(O–S)** Scanning electron micrographs of flowers and siliques. Scale bar = 500 μm. **(T)** The expression levels of *AG*, *SEP1*, *SEP2*, *SEP3*, *SEP4*, and *STK* in *35S::CsSHP Arabidopsis* flowers. Gy, gynoecium; Ov, ovule; Pl, placenta; Ca, carpel **, t-test (p < 0.01).

In *Arabidopsis*, the mature siliques in the double mutant *shp1 shp2* plant were unable to dehisce due to failure of dehiscence zone formation (Liljegren et al., 2000). To explore the function of *CsSHP*, we first transformed *CsSHP* driven by the pSUPER promoter into *shp1 shp2* mutant plants. A total of 10 transgenic plants were obtained and the T2 plants were used for further characterization. Our data showed that ectopic expression of *CsSHP* was unable to rescue the indehiscence phenotype of *shp1 shp2* mutant plant, but instead resulted in early flowering in *Arabidopsis* (**Supplementary Figures S1A, B**). Next, we transformed the *CsSHP* driven by the cauliﬂower mosaic virus 35S (CaMV 35S) into wild-type *Arabidopsis* (*35S::CsSHP/Col*). A total of 29 transgenic lines were generated. Based on *CsSHP* expression levels, three representative lines (#41, #45, #53) (T2) were chosen for further phenotypic analysis and data statistics. Compared to the wild-type control (*Col*), the expression of *CsSHP* was dramatically increased in the transgenic lines (**Supplementary Figure S2A**). Overexpression of *CsSHP* resulted in early flowering (**Figure 4A**). Quantification analysis indicated that the days to bolting was 27.2 ± 2.1 in *Col*, while that of overexpression lines varied from 21.5 ± 1.3 to 23.3 ± 1.0 (**Figure 4B**). Similarly, the number of rosette leaves upon bolting, as well as the days to the 1^st^ flower opening were significantly reduced in the transgenic lines (**Supplementary Figures S2B, C**). Moreover, ectopic expression of *CsSHP* accelerated the progression of reproductive growth in *Arabidopsis*. Under the same conditions, wild-type siliques in the main inflorescence were still green, while many of those in the transgenic plants had turned yellow and or even cracked (**Figure 4C**). Data statistics showed that the days to 1^st^ silique yellowing and the days from anthesis to silique cracking were significantly shorter in transgenic lines than *Col* plants (**Figures 4D, E**).

Much more dramatic changes were found in flower organ development in the *35S::CsSHP* transgenic lines. As compared to the wild-type control, some flower buds in transgenic plants were precociously opened (**Figures 4F, G**), flower patterning was disrupted, and the inflorescence meristem prematurely terminated (**Figures 4H, I**). For each individual flower, the flower patterning of sepal, petal, stamen, carpel was disturbed upon *CsSHP* overexpression. Some flowers in transgenic lines lack petals and stamens, consisting of only gynoecia and sepals, in which the gynoecia formed a longitudinal cleft to expose the placenta and ovules, and sepals were carpelloid (**Figure 4J**). Sometimes, one sepal of the flower was replaced by an ectopic gynoecium containing placenta tissue and ovules (**Figures 4K, L**). In more severe cases, a misshapen flower with carpelloid sepals grew from the base of another abnormal silique (**Figure 4M**), or an inflorescence grow out from the base of an ectopic carpel bearing ovules, and almost all flowers on the inflorescences were abnormal (**Figure 4N**). Scanning electron microscopy images showed that wild-type sepals were long and narrow, while that of the transgenic plants were short and round, with ectopic placenta and ovules in the inner side of the sepal (**Figures 4O, P**). Abnormal flowers with only gynoecium and carpelloid sepal grew from the inner base of another gynoecium or another flower (**Figures 4Q–S**). Statistic data showed that average of 51.6% plants displayed abnormal flowers in the transgenic lines, in which 8% of flowers were defected, as compared to none in the wild-type control (**Supplementary Figure S2D**).

Based on the carpelloid organ phenotype, a set of carpel related genes including *AG*, *SEP1/2/3/4*, and *STK* were chosen for expression analysis (Pelaz et al., 2000; Favaro et al., 2003). As compared to the wild-type control, the expression of *AG* was significantly elevated, whereas that of *SEP2*, *SEP3*, *SEP4*, and *STK* was dramatically decreased in the *CsSHP* transgenic plants (**Figure 4T**).

In addition, unlike the stretching and flat wild-type leaves, there was a considerable proportion of crooked rosette and cauline leaves curling upwards and inward in transgenic lines (**Supplementary Figure S3**).

### Interactions Of CsSHP With CsSEPs

**Figure 5:**
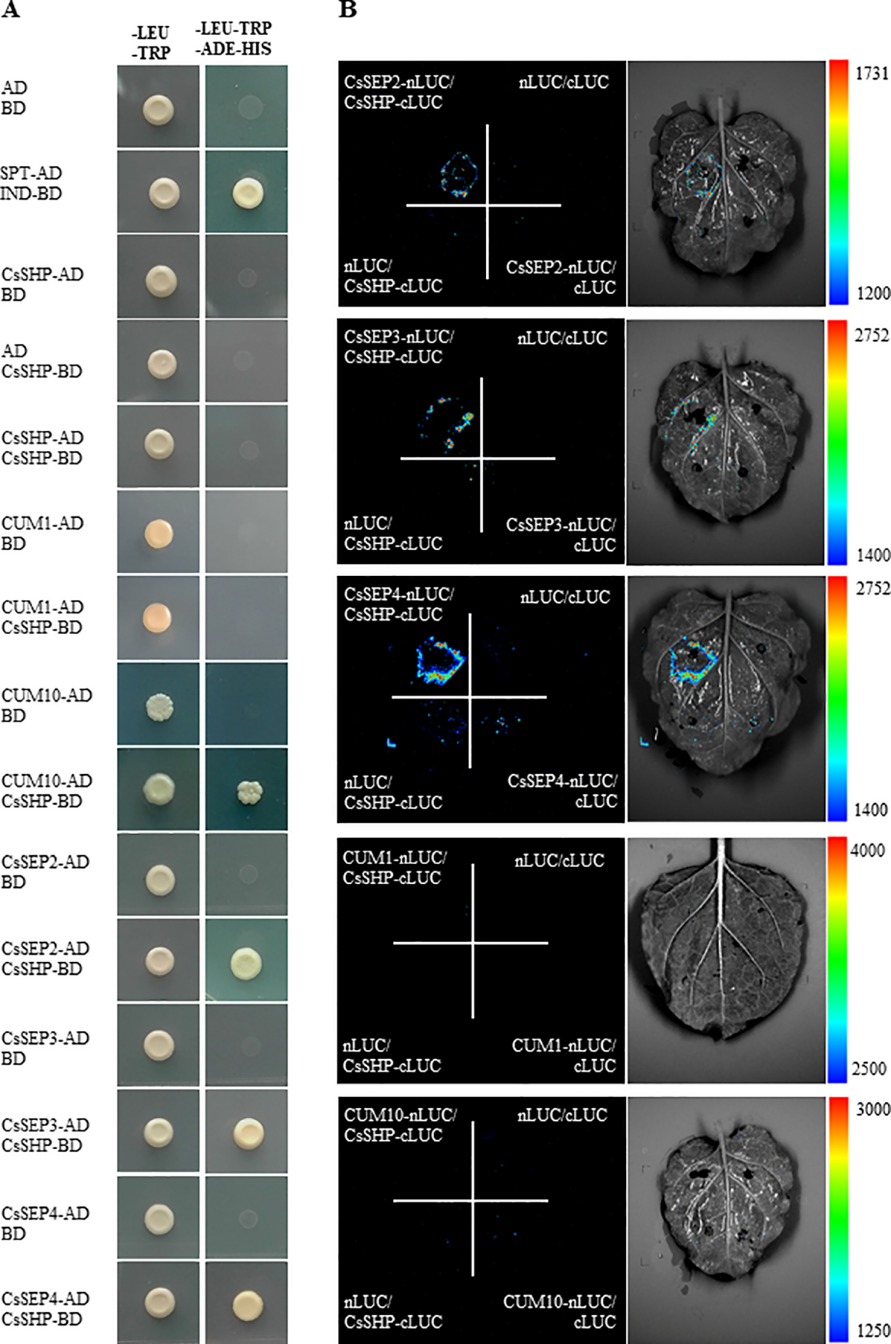
Physical interactions of CsSHP with CsSEP2, CsSEP3, and CsSEP4. **(A)** Yeast two-hybrid assay. The combination of SPT-AD and IND-BD was used as a positive control. **(B)** Luciferase (LUC) complementation imaging analysis.

*SEP1/2/3/4* are a class of organ-identity genes that are required for development of sepals, petals, stamens, and carpels in *Arabidopsis* (Pelaz et al., 2000), and SEP proteins are thought to act as a “bridge” allowing the formation of higher order complex with the floral organ identity MADS-box factors (Favaro et al., 2003). *In vitro* and *in vivo* evidence was provided for the existence of SEP, STK, and/or AG and SHP protein complexes in promoting the *Arabidopsis* ovule identity (Mendes et al., 2013). In cucumber, constitutive expression of *CUM1* (AG ortholog) resulted in sepals transformed into carpelloid structures, and petals reduced significantly in size or completely absent (Kater et al., 2001). CUM10 (STK ortholog) mediates floral organ identity in cucumber, and ectopic expression of *CUM10* resulted in partial transformation of petals into antheroid structures in petunia (Kater et al., 1998). *CsSEP2* was shown to participate in ﬂoral organ development in cucumber, since abolishment of the transcriptional activity of *CsSEP2* led to increased floral organ size with disturbed floral patterning (Wang et al., 2016). To explore the existence of possible interactions in cucumber, a yeast two-hybrid assay was performed between CsSHP and cucumber homologs of AG, SEPs, and STK. Our data showed that CsSHP displayed strong interactions with CsSEP2, CsSEP3, CsSEP4, and a weak interaction with CUM10, while CsSHP could not interact with CUM1, neither with itself to form homodimer (**Figure 5A**). To verify the protein interactions *in vivo*, a LUC complementation imaging assay was performed in the abaxial leaf epidermis of tobacco (**Figure 5B**). The reconstituted LUC enzyme can be detected by luminometer from combinations of CsSHP with CsSEP2, CsSEP3, or CsSEP4, but not from combination of CsSHP with CUM1 or CUM10, indicating that CsSHP interacts with CsSEPs to form multimeric protein complex in cucumber.

### *CsSHP* Expression Is Correlated With Fruit Maturation in Cucumber

**Figure 6:**
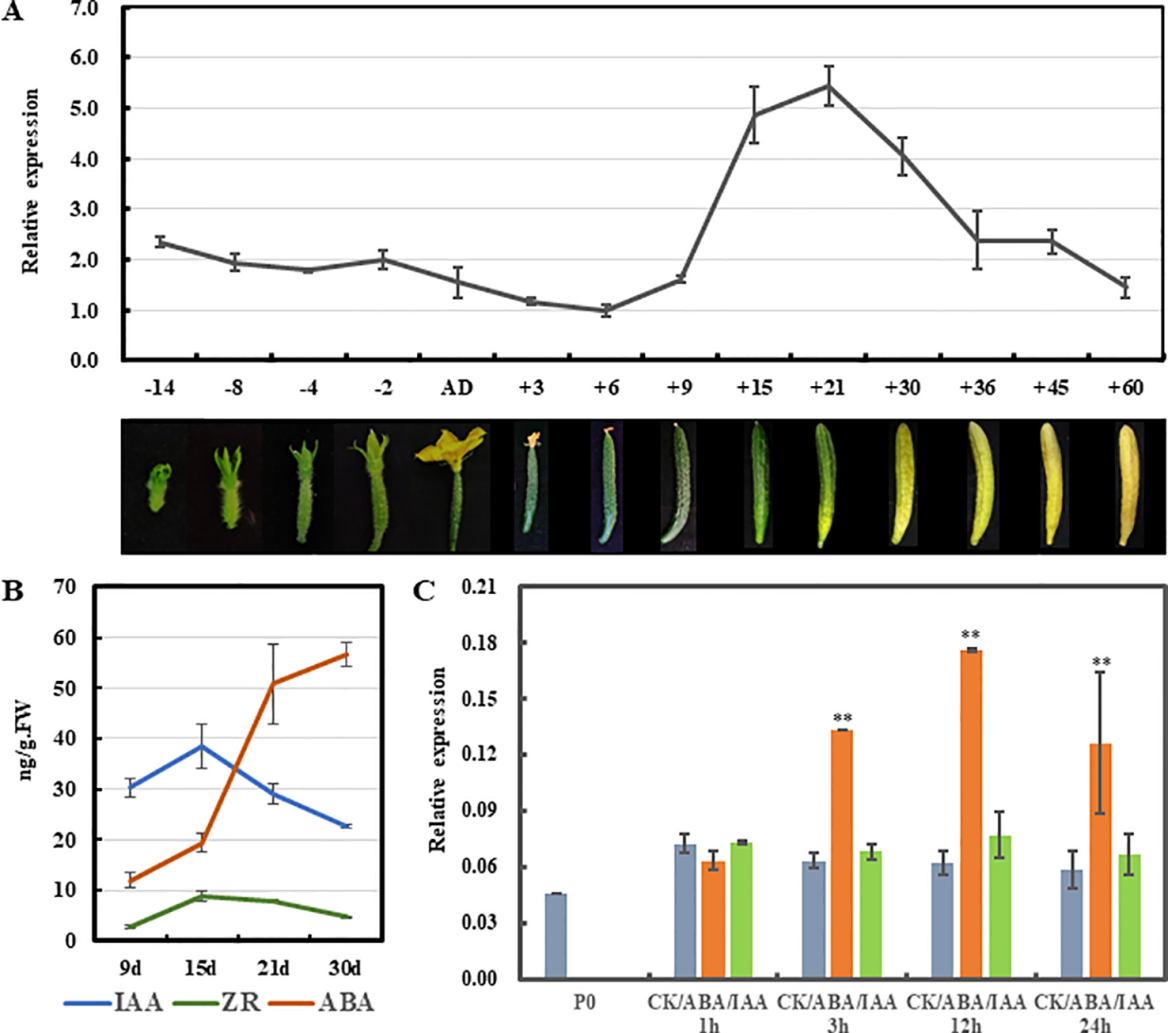
Expression analysis of *CsSHP* during fruit maturation in cucumber. **(A)** *CsSHP* expression in fruits at different developmental stages in inbred line R1461. Fruit pictures of the corresponding developmental stages are displayed at the bottom in different proportions. **(B)** Measurements of three endogenous hormones in fruit from 9 to 30 days after anthesis (DAA). Three biological replicates were performed. **(C)** Expression response of *CsSHP* to abscisic acid (ABA) or indole-3-acetic acid (IAA) treatment in cucumber ovaries of 4 days before anthesis. Three biological replicates and three technical replicates were performed **, t-test (p < 0.01).

In climacteric tomato, *TAGL1* was highly expressed in flowers at anthesis and in fruits at red ripe stage (Gimenez et al., 2010). Similarly, during the development of non-climacteric citrus fruits, the transcript levels of *CsMADS6* increased gradually, peaking at 180 DAA (breaker stage) and declining at 220 DAA (Lu et al., 2018). To understand whether *CsSHP* expression is correlated with fruit maturation in cucumber, qRT-PCR analysis was performed in cucumber fruit at fourteen developmental points. As shown in **Figure 6A**, the *CsSHP* expression showed a slight downward trend from 0.3 cm flower buds (14 days before anthesis) to commercially mature fruits (nine DAA). From 9 to 15 DAA, *CsSHP* transcripts increased threefold during the 6 days, and the highest accumulation of transcripts was detected at 21 DAA when the pericarp began to yellow. After 21 DAA, *CsSHP* expression gradually declined as fruit maturation progression and the pericarp became more and more yellow.

Cucumber is classiﬁed as a non-climacteric fruit, and endogenous ethylene displayed no inﬂuence on the postharvest yellowing of cucumber (Nilsson, 2005). However, abscisic acid (ABA) was shown to play important and direct roles in regulating cucumber fruit development and ripening. Exogenous ABA application at the turning stage promoted fruit ripening in cucumber (Wang et al., 2013). Therefore, the content of ABA, as well as the indole-3-acetic acid (IAA) and zeatin riboside (ZR), was measured in fruits of the variety R1461 from 9 to 30 DAA, when *CsSHP* expression changed drastically. Our data showed that the content of ABA increased continuously from 9 to 30 DAA as fruit ripening progression, with a dramatic upregulation from 15 to 30 DAA, while that of IAA and ZR displayed a mild increase from 9 to 15 DAA, and then decreased (**Figure 6B**), confirming that ABA may play key roles in mediating fruit ripening in cucumber. Given that the *CsSHP* expression and ABA level showed similar trend as fruit maturation, we next examined *CsSHP* response to ABA treatment in ovaries of 4 days before anthesis. Our data indicated that *CsSHP* transcription was significantly induced after ABA treatment for 3 hours, and remained at 2–3 times upregulation to 24 h (**Figure 6C**), suggesting that CsSHP may be involved in fruit maturation in cucumber through the ABA pathway.

## Discussion

MADS-box genes are critical transcription factors, participating in virtually all aspects of plant development, especially during flower and fruit development (Smaczniak et al., 2012; Wang et al., 2015). Previous study revealed the expression pattern of 43 MADS-box genes in cucumber (Hu and Liu, 2012). *CUM1*, *CUM10*, *CUM26*, *CsAP3*, and *CsSEP2* are currently known cucumber MADS-box genes that play essential roles in flower and fruit development (Kater et al., 1998; Kater et al., 2001; Sun et al., 2016; Wang et al., 2016), whereas there are still a considerable portion of MADS-box genes need to be functionally characterized. In this study, we identified the *CsSHP* gene in cucumber and revealed the roles in fruit maturation and floral organ determination.

CsSHP is a member of MADS-box family and has the conserved MADS domain and K-box domain. The MADS domain is responsible for binding downstream target DNA, and the K-box domain acts on protein-protein interactions (Shore and Sharrocks, 1995; Yang et al., 2003). The CDS length of *CsSHP* and *SHP1/2* is almost equivalent, while the genomic length of *CsSHP* gene is about twice that of *SHP1/2*, due to extremely long first and second introns (**Figure 1A**). Expression analyses by RT-PCR and *in situ* hybridization showed that *CsSHP* was specifically expressed in reproductive tissues including stamens of male flowers, stigma of female flowers, and ovaries (**Figure 3**). Such expression is similar to that of *TAGL1* in tomato and *NbSHP* in tobacco, but different from *SHP2* in *Arabidopsis*, which is specifically expressed in gynoecium and absent in stamen (Vrebalov et al., 2009; Gimenez et al., 2010; Fourquin and Ferrandiz, 2012). Interestingly, GUS signals driven by *CsSHP* promoter were detected only in stamens and valve margin in *Arabidopsis*, but not in developing gynoecium (**Figure 3**). There are two possible reasons for such discrepancy. One is the different flower structure in *Arabidopsis* (complete flower) and cucumber (unisexual flower), and the other is that the promoter sequence used for driving the GUS signal was unable to confer the correct expression pattern of *CsSHP*. Considering that the second intron of AG/PLE genes contain multiple regulatory elements that determine the proper spatial and temporal expression (Liu and Liu, 2008; Gu et al., 2018), it is more plausible that the expression pattern of *CsSHP* is coordinately controlled by its promoter and introns.

Overexpression of *CsSHP* in wild-type *Arabidopsis* resulted on ectopic carpelloid organs with the characteristics of gynoecium: stigma-like tissue at the top, placenta tissue in the middle, and infertile ovules along the valve margin (**Figure 4**). The inflorescence or flower patterning were often terminated in an ectopic carpel, showing a phenotype of “flower in carpel” or “inflorescence in carpel” (**Figure 4**). Although some phenotypes like early ﬂowering, curly leaves and prematurely open ﬂower buds, are similar to those previously described upon overexpression of *SHP* orthologs, such as *Arabidopsis SHP1/2* and tomato *TAGL1*, in *Arabidopsis* (Favaro et al., 2003; Vrebalov et al., 2009), the carpelloid phenotype caused by *CsSHP* overexpression is more severe. For example, *35S::SHP1/2* in *Arabidopsis* showed a transformation of petals toward stamens and a partial conversion of sepals toward carpels, with stigmatic papillae on it (Favaro et al., 2003). However, in the *Arabidopsis* inflorescences of *35S::CsSHP* plants, ectopic carpels may grow anywhere of the flower (**Figure 4**). The carpelloid phenotype in *35S::CsSHP* transgenic plants suggested the function of *CsSHP* in specifying carpel identity in cucumber. Consistently, the expression of *AG* was significantly elevated, while that of STK was almost abolished in the transgenic flowers (**Figure 4**), which may due to the ectopic carpelloid organs with infertile ovules. Interestingly, the expression of *SEP2*, *SEP3* and *SEP4* was dramatically decreased in the *35S::CsSHP* transgenic flowers (**Figure 4**). However, without a time course analysis in *CsSHP* inducible lines, we were unable to differentiate the expression change of *CsSEP2/3/4* is a cause or a result of floral patterning defect. Biochemical data showed that CsSHP interacts with CsSEP2/3/4 at protein level (**Figure 5**). Loss of *CsSEP2* function led to disturbed flower patterning with enlarged floral organs in cucumber (Wang et al., 2016). Therefore, we hypothesized that CsSHP may specify carpel identity in cucumber through interacting with CsSEP2 to form protein complex. In fact, SHP homologs were shown to interact with SEP-like proteins in *Arabidopsis*, tomato, and soybean (Favaro et al., 2003; Leseberg et al., 2008; Mendes et al., 2013; Chi et al., 2017), indicating the interactions between SHP and SEPs are relatively conserved among species.

Cucumber fruit is harvested immature and consumed freshly or as processed pickles. Fruit ripening is important for seed maturation, but has adverse effect on cucumber production and post-harvest shelf life. Unlike the dry pod in *Arabidopsis*, cucumber fruit has no cracking characteristics, and fruit ripening involved in changes of color, texture, and aroma (Seymour et al., 2008). As a non-climacteric fruit, ethylene has no inﬂuence on the postharvest yellowing of cucumber (Nilsson, 2005), but instead, ABA application at the turning stage promoted fruit ripening (Wang et al., 2013). Here, we found that ectopic expression of *CsSHP* resulted in early flowering and accelerated ripening in *Arabidopsis* (**Figure 4**). Moreover, *CsSHP* expression is significantly increased during the fruit yellowing from 9 to 21 DAA, concomitant with the dramatic increase of ABA level (**Figure 6**), implying the positive roles of *CsSHP* in cucumber ripening. Further, *CsSHP* expression was found to be induced upon exogenous ABA application (**Figure 6D**). Hence, it is plausible to speculate that high level of ABA from commodity fruit stage (nine DAA) induces *CsSHP* expression, which positively modulates carotenoid metabolism and ripening-related genes, just like AGL1 in tomato and CsMADS6 in citrus (Vrebalov et al., 2009; Lu et al., 2018), to promote fruit yellowing and fruit ripening in cucumber. Further studies are needed to test above hypothesis using transgenic cucumbers through CRISPR/Cas9 techniques (Hu et al., 2017).

## Data Availability Statement

All datasets generated for this study are included in the article/**Supplementary Material**.

## Author Contributions

ZC and XZ conceived this project. ZC, SZ, XL, GC, ZW, RG, JS, WS performed the experiments, ZC and XZ wrote the manuscript, XL, ZZ and DH contributed to critical discussions. All authors read and approved the final version.

## Funding

This study was supported by the National Key Research and Development Program [2018YFD1000800], National Natural Science Foundation of China [31572132] and [31772315], and the Construction of Beijing Science and Technology Innovation and Service Capacity in Top Subjects [CEFFPXM2019_014207_000032].

## Conflict of Interest

The authors declare that the research was conducted in the absence of any commercial or ﬁnancial relationships that could be construed as a potential conﬂict of interest.
