# Supplementary material for: The MADS-Box Gene CsSHP Participates in Fruit Maturation and Floral Organ Development in Cucumber
Source: Front Plant Sci. 2020 Feb 10;10:1781. doi: 10.3389/fpls.2019.01781 (PMC7025597; doi:10.3389/fpls.2019.01781)
Supplement: Supplementary file 1 [file DataSheet_1.pdf]

The MADS-box gene *CsSHP* participates in fruit maturation and floral organ development in cucumber

Zhihua Cheng[1], Shibin Zhuo[1], Xiaofeng Liu[1], Gen Che[1], Zhongyi Wang[1], Ran Gu[1], Junjun Shen[1], Weiyuan Song[1], Zhaoyang Zhou[1], Deguo Han[2,*] and Xiaolan Zhang[1,*]

> CsSHP transcript sequence

ATGGGAAGAGGTAAGATTGAAATAAAAAGAATTGAAAATACTACAAATCGTC
AAGTCACCTTTTGCAAGAGAAGAAATGGATTGCTTAAAAAAGCTTATGAGCT
GTCTGTTCTTTGCGATGCTGAAGTTGCTCTCATCGTCTTCTCTACTCGTGGTCG
TCTCTACGAATACGCAAATAATAGTGTTAGAGGAACGATTGAGAGGTACAAGA
AAGCATTTGCTGATTCTTCCAATTCCGGATTATCAGTTGCCGAAGCTAATGTAC
AGTTTTACCAACAAGAAGCCACCAAGTTGAAGAGACAGATTAGGGAAATTCA
GAACTCAAACAGGCATATCCTGGGAGAAGCACTCAGCTCATTGCCATTAAAA
GAGCTCAAAAGTCTTGAGGGCAGATTGGAGAGAGGTATCAGCAAAGTTAGG
GCTAAAAAGAACGAAACCTTGTTTGCAGAAATGGAATTCATGCAAAAAAGGG
AAATGGAACTTCAGAGCCACAATAACTATCTGAGAACACAGATTGCAGAACA
CGAAAGAATACAACAGCAGCAGCAGCAACAACAGCAAACGAACATGATGCA
AAGGGCAACATATGAGAGTGTGGGAGGGCAATATGATGATGAGAATAGAAGT
ACTTATGGGGCTGTAGGGGCGCTTATGGATTCAGACAGCCATTATGCTCCTCA
AGACCATCTCACTGCCCTTCAGCTTGTTTAA

> CsSHP amino acid sequence

MGRGKIEIKRIENTTNRQVTFCKRRNGLLKKAYELSVLCDAEVALIVFSTRGRLY
EYANNSVRGTIERYKKAFADSSNSGLSVAEANVQFYQQEATKLKRQIREIQNSNR
HILGEALSSLPLKELKSLEGRLERGISKVRAKKNETLFAEMEFMQKREMELQSH
NNYLRTQIAEHERIQQQQQQQQTNMMQRATYESVGGQYDDENRSTYGAVGA
LMDSDSHYAPQDHLTALQLV

Supplementary S1 Transcript sequence of GmAGL1 and its deduced peptide sequence.

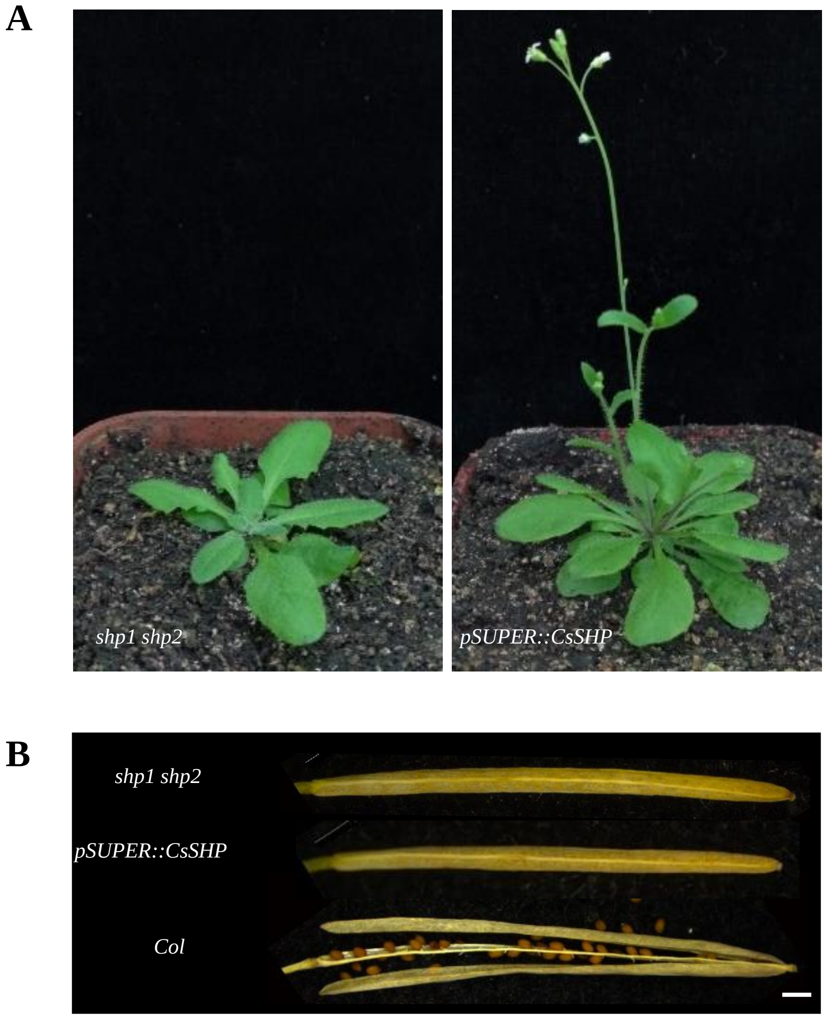

Supplementary Fig S1 Ectopic expression of *CsSHP* in *shp1shp2* mutant *Arabidopsis*. (A) *CsSHP* promoted flowering in *shp1 shp2* mutant. (B) Silique phenotypes of *CsSHP* transgenic lines, *shp1 shp2* mutant and wild-type *Arabidopsis*. Scale bar = 1 mm.

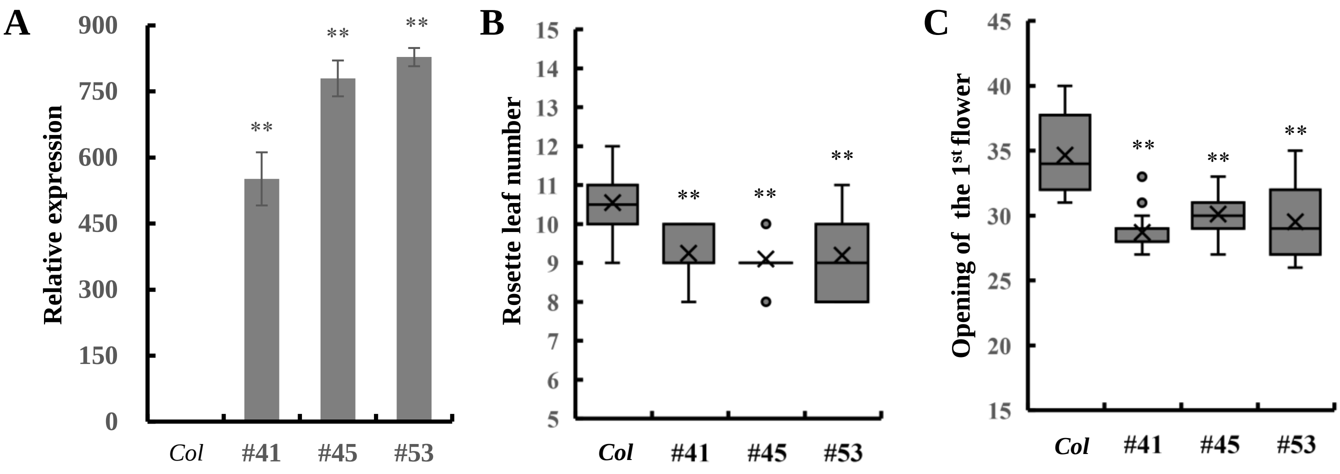

D

Plant type	Plants with abnormal flowers/Total plants	Abnormal flowers/Total flowers
Col	0/5	0/194
#41	14/20	56/640
#45	11/20	37/609
#53	6/20	31/293

Supplementary Fig S2 Quantifications of the flowering phenotype in *35S::CsSHP* transgenic lines in *Arabidopsis*. (A) Expression analysis of *CsSHP* in *Col* and transgenic lines. Three biological replicates and three technical replicates were performed (B) Box-blot of the number of rosette leaf to bolting. (C) Box-plot of the days to opening of the 1st flower. (D) Quantification of the abnormal flowers in transgenic lines. The data were the average of 20 plants for each line.

A

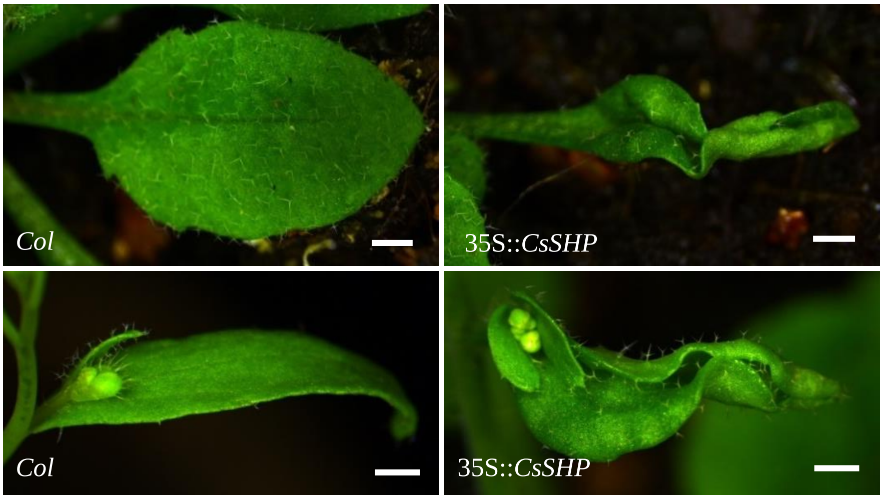

B

Plant type	Plants with abnormal rosette leaf/Total plants	Plants with abnormal cauline leaf/Total plants
Col	0/20	0/20
**#41**	16/20	6/20
**#45**	17/19	3/19
**#53**	15/19	8/19

Supplementary Fig S3 Leaf phenotype in *35S::CsSHP* transgenic plants in *Arabidopsis*. (A) Leaf morphology in transgenic lines. Top row: Rosette leaf; bottom row: Cauline leaf. Scale bar = 1 mm. (B) Quantification of abnormal leaf in transgenic lines. The data were the average of 20 plants for each line.

Supplementary Table S1 Gene information used in this study

Gene name	Species	Accession number
CsSHP	*Cucumis sativus*	NP_001292697.1
CUM1	*Cucumis sativus*	NP_001292633.1
CsSEP2	*Cucumis sativus*	NP_001267667.1
AtAG	*Arabidopsis thaliana*	NP_567569.3
AtSHP1	*Arabidopsis thaliana*	NP_001190130.1
AtSHP2	*Arabidopsis thaliana*	NP_850377.1
SEP1	*Arabidopsis thaliana*	NP_001119230.1
SEP2	*Arabidopsis thaliana*	NP_186880.1
SEP3	*Arabidopsis thaliana*	NP_564214.2
SEP4	*Arabidopsis thaliana*	NP_178466.1
STK	*Arabidopsis thaliana*	NP_001078364.1
TAG1	*Solanum lycopersicum*	NP_001266181.1
TAGL1	*Solanum lycopersicum*	AAM33101.2
RIN	*Solanum lycopersicum*	NP_001233976.1
FAR	*Antirrhinum majus*	CAB42988.1
PLE	*Antirrhinum majus*	AAB25101.1
pMADS3	*Petunia hybrida*	Q40885.1
FBP6	*Petunia hybrida*	CAA48635.1
GmAGL1	*Glycine max*	APX55173.1
VvMADS1	*Vitis vinifera*	NP_001268105.1
STAG1	*Fragaria ananassa*	AAD45814.1
FaSHP	*Fragaria ananassa*	AGU92563.1
CitMADS61	*Citrus unshiu*	XP_006449577.1
CitMADS6	*Citrus unshiu*	XP_024041419.1
BnSHP1	*Brassica napus*	AAK00646.1
PpPLE	*Prunus persica*	XP_007217264.1
ClSHP	*Citrullus lanatus*	ClCG02G018350

Supplementary Table S2 Primer information used in this study

**Primers for *CsSHP***	
CsSHP-clone-F	ATGGGAAGAGGTAAGATTGA
CsSHP-clone-R	TTAAACAAGCTGAAGGGCAGT
CsSHP-121-F	GCTCTAGAATGGGAAGAGGTAAGATTGA
CsSHP-121-R	TCCCCCGGGTTAAACAAGCTGAAGGGCAGT
CsSHP-1300-F	ATCGACTCTAGAAAGCTTATGGGAAGAGGTAAGATT
CsSHP-1300-R	GGTACCGGATCCACTAGTAACAAGCTGAAGGGCAGT
CsSHP-promoter-F	CATGATTACGCCAAGCTT CTCAAAGTAGCTCACAAATTAAGG
CsSHP-promoter-R	ACTGACCACCCGGGGATCCTTTTTTTTGGGAAGAGGCAGATCC
**Primers for qRT-PCR**	
CsSHP-Q-F	GTGTGGGAGGGCAATATGATGAT
CsSHP-Q-R	CTTGAGGAGCATAATGGCTGTCTG
UBI-Q-F	CACCAAGCCCAAGAAGATC
UBI-Q-R	TAAACCTAATCACCACCAGC
Actin2-Q-F	CCTTCGTCTTGATCTTGCGG
Actin2-Q-R	AGCGATGGCTGGAACAGAAC
SEP1-Q-F	TTCTTGGGGAGGATTTAGGACC
SEP1-Q-R	TGTGTCTTGATGGACCGAACTT
SEP2-Q-F	TCTCTGCGATGCTGAAGTCTCT
SEP2-Q-R	CTCCAAGAAGATTTCTCTGCTGAC
SEP3-Q-F	TACAAAGGAGCTTGAGTCAC
SEP3-Q-R	CCTCTTCTTGGTTAGGGTTC
SEP4-Q-F	GGTATGGCGAGGACGGTTGA
SEP4-Q-R	GCTCTTCACCTAGCAAATGCCT
AG-Q-F	ATAATCAGCATACAAAACTCCAAC
AG-Q-R	ATACTTCTCTCTAATCTGCCTTCC
STK-Q-F	CAACAGGAATCTGATGGGAGACT
STK-Q-R	TGATACCTCTCCACTTCTGCTACC
**Primers for *in situ* probes**	
CsSHP-SP6	GATTTAGGTGACACTATAGAATGCTGATTGAGAGGTACAAGAAAG
CsSHP-T7	TGTAATACGACTCACTATAGGGTTAAACAAGCTGAAGGGCA

**Primers for yeast two-hybrid assay**	
CsSHP-AD/BK-F	CGGGATCCTGATGGGAAGAGGTAAGATTGA
CsSHP-AD-R	GCCTCGAGTTAAACAAGCTGAAGGGCAGT
CsSHP- BK-R	AACTGCAGTTAAACAAGCTGAAGGGCAGT
CUM10-AD-F	CGGGATCCTGATGAACTCTCTGTTCTTTGCTGAAG
CUM10-AD-R	GCCTCGAGTCACCCAAGATGAAGCATTTTCT
CsSEP2-AD-F	CCGGAATTCCGGATGCTTAAAACGCTGGAGAGG
CsSEP2-AD-R	CGGATCCGTCAAAGCATCCAACCAGGGAG
CsSEP3-AD-F	CCGGAATTCCGGATGCTCAAAACTCTAGAGAGATA
CsSEP3-AD-R	CGGATCCGTCAAGGCAACCATCCTGGCAG
CsSEP4-AD-F	CATATGATGAATTCTCAAGCTCTCCCAGGCATTA
CsSEP4-AD-R	CGGATCCGTCAAAGCAGCATCCAATGTGAA
**Primers for LUC complementation imaging assay**	
CsSHP-C-F	TCGTACGCGTCCCGGGGCATGGGAAGAGGTAAGATT
CsSHP-C-R	GTCCATTTGTTGGATCCGAACAAGCTGAAGGGCAG
CUM10-N-F	CGAGCTCGGTACCCGGGATCCATGAACTCTCTGTTCTTT
CUM10-N-R	CGCGTACGAGATCTGGTCGACCCCAAGATGAAGCATTT
CsSEP2-N-F	CGAGCTCGGTACCCGGGATCCATGCTTAAAACGCTGGA
CsSEP2-N-R	CGCGTACGAGATCTGGTCGACAAGCATCCAACCAGGGA
CsSEP3-N-F	CGAGCTCGGTACCCGGGATCCATGCTCAAAACTCTAGAG
CsSEP3-N-R	CGCGTACGAGATCTGGTCGACAGGCAACCATCCTGGCAG
CsSEP4-N-F	CGAGCTCGGTACCCGGGATCCATGAATTCTCAAGCTCT
CsSEP4-N-R	CGCGTACGAGATCTGGTCGACAAGCAGCATCCAATGTG
